# Improving the Push-Out Bond Strength of Fiber Posts in Diabetic Dentin: The Role of Chlorexidine Irrigation and Resin Cements

**DOI:** 10.3390/jfb16010004

**Published:** 2024-12-25

**Authors:** Beyza Arslandaş Dinçtürk, Arzu Şahin Mantı, Cemile Kedici Alp, Ayşenur Altuğ Yıldırım, Arzu Kaya Mumcu

**Affiliations:** 1 Restorative Dentistry Department, Faculty of Dentistry, Gazi University, 06490 Ankara, Türkiye; beyzaarslandas@gazi.edu.tr (B.A.D.); aysenuraltug@gazi.edu.tr (A.A.Y.); 2 Department of Endodontics, Faculty of Dentistry, Gazi University, 06490 Ankara, Türkiye; arzumanti@gazi.edu.tr; 3 Department of Endodontics, Faculty of Dentistry, Kütahya Health Sciences University, 43020 Kütahya, Türkiye; arzu.kayamumcu@ksbu.edu.tr

**Keywords:** diabetes mellitus, fiber post, resin-based luting agents, push-out bond strength, chlorhexidine, root canal dentin

## Abstract

This study evaluated the effect of resin cements and post-space irrigation solutions on the push-out bond strength of diabetic and non-diabetic dentin. A total of 160 human central teeth (80 diabetic, 80 non-diabetic) were prepared using X5 files and obturated with AH Plus sealer and X5 gutta-percha. Post spaces were prepared, and teeth were divided into eight groups based on resin cements (Variolink N, Panavia SA Universal) and irrigation protocols (saline, saline + 2% CHX). A 1 mm slice from each tooth’s middle third was tested for push-out bond strength. Statistical analysis was performed using three-way ANOVA and Tukey HSD tests. In non-diabetic dentin, saline + CHX with Variolink N achieved the highest bond strength. In diabetic dentin, saline with Panavia SA Universal resulted in the lowest bond strength (*p* < 0.05). The dentin type, resin cement, and irrigation solution significantly impacted fiber post bond strength, and CHX irrigation improved it.

## 1. Introduction

Diabetes mellitus (DM) is a chronic metabolic disorder characterized by impaired insulin secretion or activity in peripheral tissues, resulting in persistent hyperglycemia [1]. This systemic condition affects various body tissues, including dental tissues such as dentin and enamel, leading to an increased prevalence of dental caries, oral infections, and tooth loss in diabetic patients compared to non-diabetic individuals [2]. Systemic hyperglycemia results in significant local changes, including alterations in the dental pulp, odontoblast activity, and dentinal tubule density, which can adversely affect the structural and mechanical properties of dentin [3,4].

The bond strength between restorative materials and dental tissues is one of the critical factors determining the long-term success of dental restorations [5]. Resin cements and adhesive systems are widely used in restorative dentistry because of their reliable adhesive and mechanical properties [6]. The bonding process is based on acid etching, which demineralizes the dentin surface and exposes the collagen matrix, allowing resin adhesives to penetrate and form a hybrid layer. This hybrid layer acts as a stress-absorbing interface during cyclic loading, which is essential to ensure the durability and stability of bonded restorations. However, incomplete resin infiltration into the collagen matrix can leave unprotected collagen fibrils, rendering the hybrid layer susceptible to enzymatic degradation and failure [7].

In diabetic patients, hyperglycemia and oxidative stress are known to upregulate matrix metalloproteinases (MMPs), particularly MMP-2 and MMP-9 [8]. These enzymes degrade the exposed collagen network within the hybrid layer, resulting in its fragmentation and weakening the resin–dentin bond over time [9]. Furthermore, it has been reported that patients with diabetes mellitus (DM) exhibit higher concentrations of MMP-8 in their saliva, which is thought to enhance enzymatic activity and contribute to the weakening of the dentin–resin interface [10]. However, there is limited information in the literature regarding the effects of DM on the interface between dentin and adhesive [9,11]. In addition, diabetes can alter the oral environment by lowering salivary pH and increasing bacterial plaque accumulation, which can exacerbate microleakage and predispose restorations to recurrent caries [12].

Studies have shown that diabetic dentin exhibits significant structural and physicochemical changes compared to healthy dentin [9,13]. For example, studies evaluating the push-out bond strength of mineral trioxide aggregate (MTA) to root canal dentin found a significant reduction in bond strength in diabetic dentin, which was attributed to increased tubule density and reduced peritubular dentin formation [4]. These structural changes may compromise the adhesion of restorative materials and require specific strategies to optimize bond performance.

Fiber posts are often used in endodontically treated teeth to improve the retention and reinforcement of restorations [14]. The cementation of fiber posts with adhesive systems has been shown to improve post retention, reduce microleakage, and minimize bond failure at the dentin–fiber post interface [15]. Two primary adhesive strategies are used: (1) separate-etch adhesive systems, which involve multiple steps of etching, priming, and bonding, and (2) self-adhesive systems, which simplify the procedure by combining etching and bonding into a single step without the need for rinsing [16]. While self-adhesive systems are less technique-sensitive, studies have shown that separate-etch adhesives generally achieve higher bond strength due to their superior hybrid layer formation [16].

Chlorhexidine (CHX) is a commonly used irrigation solution in endodontic treatment due to its broad and prolonged antimicrobial activity and biocompatibility [17]. It has also been extensively studied in the literature for its role in stabilizing the hybrid layer by reducing the activity of collagen-degrading matrix metalloproteinases in radicular dentin. By inhibiting MMP activity in the dentin matrix, CHX preserves the integrity of collagen fibrils and increases the durability of the resin–dentin bond [18]. Cecchin et al. showed that CHX pretreatment significantly improved the bond strength of resin-cemented fiber posts in healthy teeth [19]. Despite these promising findings, to our knowledge, there are currently no published data on the efficacy of CHX in improving bond strength in diabetic dentin, where collagen degradation is exacerbated by hyperglycemia-induced MMP activation.

Given the structural and biochemical changes in diabetic dentin and the limited evidence regarding the role of CHX in this context, further investigation is warranted. Therefore, the aim of this study was to evaluate the effects of different resin cements and CHX irrigation on the push-out bond strength of fiber posts to root canal dentin in diabetic and non-diabetic teeth. The null hypothesis was that the type of resin cement and the use of CHX irrigation would not significantly affect the push-out bond strength of fiber posts in diabetic dentin compared to non-diabetic dentin.

## 2. Materials and Methods

The materials used in this study and their compositions are presented in Table 1.

### 2.1. Tooth Selection

This study was approved by the Ethics Committee of Kutahya Health Sciences University (ethical protocol no. 2024/13-01).

In this study, 160 single-rooted human central teeth were selected, all with a minimum root length of 17 mm and no curvatures, which had been extracted due to periodontal or orthodontic reasons. The canal diameters at the cervical region of these teeth exceeded the post diameters by 0.1–0.3 mm. In total, 80 teeth were extracted from patients with DM and 80 teeth were extracted from patients without diabetes mellitus (80 diabetic, 80 non-diabetic). Following decoronation, the roots were standardized to a length of 17 mm and stored in distilled water at 37 °C until the experimental procedures commenced.

### 2.2. Root Canal Treatment

The root canals of teeth were prepared to the same apical size using X5 files (ProTaper Next, Dentsply Maillefer, Ballaigues, Switzerland). During the instrumentation process, irrigation was performed with 5 mL of 2.5% NaOCl solution (Microvem, İstanbul, Türkiye) following a standardized protocol.

Following the final irrigation with 3 mL of NaOCl, 17% EDTA was applied for 1 min, and then the canals were rinsed with saline for 1 min. The canals were then dried using X5 paper points (ProTaper, Dentsply Maillefer) and obturated using the cold lateral compaction technique with a sealer (AH Plus, Dentsply Maillefer) and X5 gutta-percha (Dentsply Maillefer). The specimens were stored at 37 °C and 100% humidity for one week to ensure the complete setting of the filling materials.

The apical diameter of the prepared post space was set at 0.8 mm, while the coronal diameter measured approximately 1.5 mm. The fiber post tapered from 0.5 mm at the apical end to 1.2 mm at the coronal end, creating a cement layer thickness of approximately 0.3 mm within the root canal.

Subsequently, the roots were divided into eight groups based on post-space irrigation protocols and fiber post (Cytec Blanco, Hahnenkratt, Königsbach-Stein, Germany) cementation procedures.

Group 1: In non-diabetic dentin, post spaces were irrigated with 5 mL saline for 3 min and dried with paper points (Dentsply Maillefer, Ballaigues, Switzerland), and fiber posts (Cytec Blanco, Hanner-Kratt, Germany) were cemented with Variolink N (Ivoclar Vivadent, AG, Schaan, Liechtenstein). The post spaces were treated according to the manufacturer’s instructions explained in Table 1. Fiber posts were cemented with Variolink N and polymerized for 20 s using an LED (D-Light Pro, GC, Leuven, Belgium).

Group 2: In non-diabetic dentin, post spaces were irrigated with 5 mL saline solutions for 1.5 min, followed by 5 mL CHX solutions for 1.5 min and dried with paper points, and fiber posts (Cytec Blanco, Hanner-Kratt, Germany) were cemented similarly to Group 1.

Group 3: In non-diabetic dentin, post spaces were irrigated with 5 mL saline for 3 min, and fiber posts (Cytec Blanco, Hanner-Kratt, Germany) were cemented with self-adhesive resin cement (Panavia SA Universal, Kuraray Noritake Dental) without any pretreatment and polymerized with an LED light curing unit (D-Light Pro, GC, Leuven, Belgium) for 20 s.

Group 4: In non-diabetic dentin, post spaces were irrigated as in Group 2 and fiber posts were cemented with Panavia SA Universal, similarly to Group 3.

Group 5: In diabetic dentin, post spaces were irrigated with 5 mL saline for 3 min and dried with paper points, and fiber posts were cemented similarly to Group 1.

Group 6: In diabetic dentin, post spaces were irrigated as in Group 2 and fiber posts were cemented similarly to Group 1.

Group 7: In diabetic dentin, post spaces were irrigated with 5 mL saline for 3 min and dried with paper points, and fiber posts were cemented with Panavia SA Universal similarly to Group 3.

Group 8: In diabetic dentin, post spaces were irrigated as in Group 2 and fiber posts were cemented with Panavia SA Universal similarly to Group 3.

### 2.3. Evaluation of Push-Out Bond Strength

**Figure 1:**
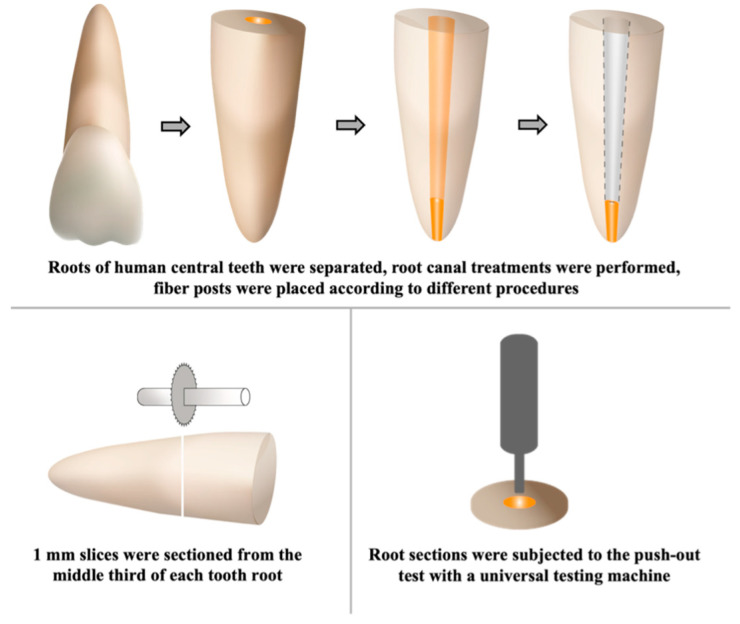
Flow chart.

After soaking in distilled water at 37 °C for 24 h, a 1 mm thick slice was sectioned from the middle third of each tooth root, perpendicular to the long axis of the root, using a low-speed diamond saw with water cooling (Isomet 1000; Buehler, IL, USA). The force was divided by the bonded surface area of the post segment to evaluate push-out bond strength values in Mpa (Figure 1).

Specimens from the roots were subjected to a push-out test using a universal test machine ((Shimadzu AG-IS Autograph, Shimadzu Scientific Instruments, Marlborough, MA, USA) Instron Corp.) at a crosshead speed of 0.5 mm/min, until displacement occurred. A 4 mm long tip with a diameter of 0.6 mm was employed for the push-out test, and the forces were applied in the apical-to-coronal direction.

Failure types were evaluated using a microscope operating at 25× magnification. Failure types were classified as follows: adhesive failure between fiber post and cement (type 1), adhesive failure between dentin and cement (type 2), or mixed (type 3).

### 2.4. Field-Emission Scanning Electron Microscopy Analysis (FE-SEM)

After the solutions used for each group were applied as described previously, all samples were dried in a desiccator for 12 h and sputter-coated with gold in a vacuum coating device (Leica Ace 200, Leica Microsystems, Wetzlar, Germany). Microphotographs of some surface areas were taken at 2500× magnification.

### 2.5. Statistical Analysis

Statistical analyses performed to assess the study’s findings were conducted using the IBM SPSS Statistics 22 program. Kolmogorov–Smirnov and Shapiro–Wilk tests were used to assess the study data’s suitability for normal distribution, and the results showed that the parameters were normally distributed. To assess the combined impact of variations in tooth tissue, variations in resin cements, and variations in post-space irrigation solutions on bond strength, a three-way ANOVA test and a post hoc Tukey HSD test were used. Significance level was set at *p* < 0.05.

## 3. Results

**Figure 2:**
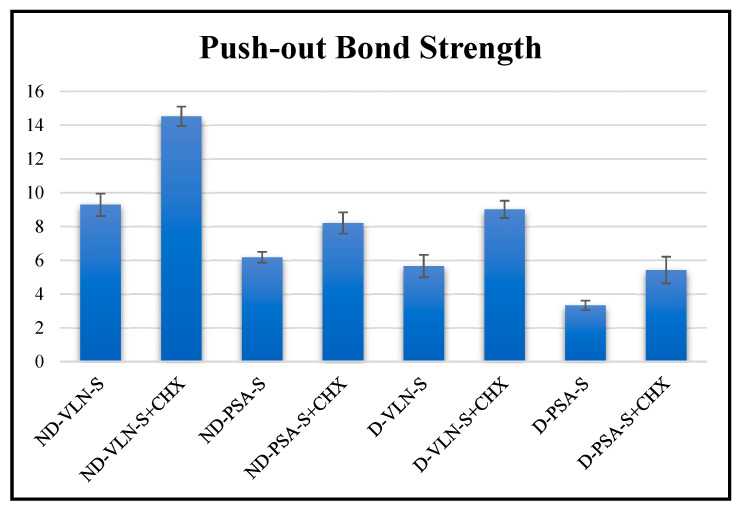
Evaluation of bond strength (ND: non-diabetic, D: diabetic, PSA: Panavia SA Universal, VLN: Variolink N, S: saline, CHX: chlorhexidine).

Regarding bond strength, there was a statistically significant difference (*p*: 0.001; *p* < 0.05) between groups. Following the use of the post hoc Tukey HSD test to identify which groups differed significantly, Group 2’s (non-diabetic, Variolink N, Saline + CHX; Figure 4b) bond strength was found to be significantly higher than that of all other groups (*p* < 0.05). Group 5’s (diabetic, Panavia SA Universal, Saline; Figure 4g) bond strength was notably less strong than that of all other groups (*p* < 0.05) (Figure 2).

When Variolink N luting cement was used, the bond strength of non-diabetic dentin (Figure 4b) was statistically significantly higher than that of diabetic dentin (Figure 4f) irrigated with saline solution and saline + CHX solution (*p*: 0.001; *p* < 0.05). In addition, when Panavia SA Universal luting cement was used, the bond strength of non-diabetic dentin (Figure 4d) was statistically significantly higher than that of diabetic dentin (Figure 4h) irrigated with saline solution and saline + CHX solution (*p*: 0.001; *p* < 0.05). When Variolink N luting cement was used, the bond strength of irrigation with saline + CHX solution was statistically significantly higher than irrigation with saline in both non-diabetic and diabetic dentin (*p*: 0.001; *p* < 0.05). Furthermore, when Panavia SA Universal luting cement was used, the bond strength of irrigation with saline + CHX solution was statistically significantly higher than irrigation with saline in both non-diabetic and diabetic dentin (*p*: 0.001; *p* < 0.05). The bond strength of Variolink N luting cement was statistically significantly higher than Panavia SA Universal luting cement in both saline solution and saline + CHX solution groups at final irrigation in non-diabetic and diabetic dentin (*p*: 0.001; *p* < 0.05) (Table 2).

**Figure 3:**
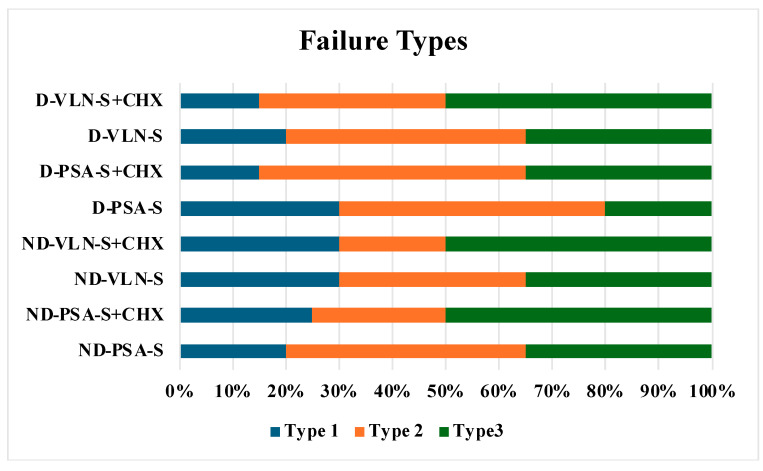
Failure type analysis (ND: non-diabetic, D: diabetic, PSA: Panavia SA Universal, VLN: Variolink N, S: saline, CHX: chlorhexidine. Type 1: adhesive failure between fiber post and cement, Type 2: adhesive failure between dentin and cement, Type 3: mixed).

Failure types classified as adhesive failure between fiber post and cement (type 1), adhesive failure between dentin and cement (type 2), and mixed failure (type 3) are shown in Figure 3. The highest percentage of the mixed type was observed in groups 2, 4, and 6 (50%), with adhesive failure between post and cement in groups 1, 2, 7 (30%) and adhesive failure between dentin and cement in groups 7 and 8 (50%).

**Figure 4:**
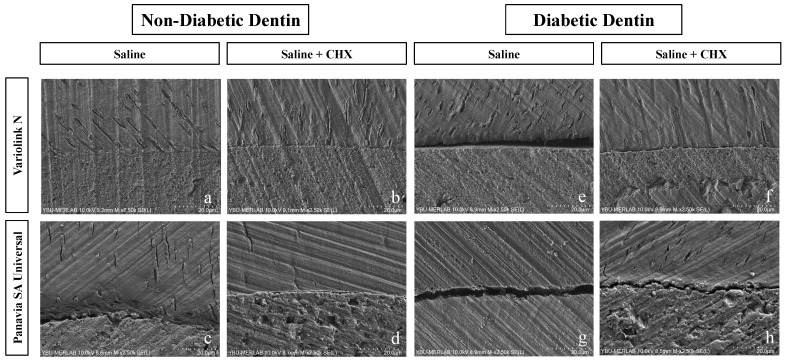
FE-SEM images of resin cements (Variolink N, Panavia SA Universal) applied to different dentin surfaces (non-diabetic, diabetic) with different post-space irrigations (saline, saline + CHX). ((**a**) ND, VLN, S; (**b**) ND, VLN, S + CHX; (**c**) ND, PSA, S; (**d**) ND, PSA, S + CHX; (**e**) D, VLN, S; (**f**) D, VLN, S + CHX; (**g**) D, PSA, S; (**h**) D, PSA, S + CHX) (ND: non-diabetic, D: diabetic, PSA: Panavia SA Universal, VLN: Variolink N, S: saline, CHX: chlorhexidine).

FE-SEM images were evaluated to determine the bonding between dentin and resin cements and fiber posts. When bonding areas were examined, a smoother and continuous image was observed in the groups with high bond strength, while spacing was observed in the groups with low bond strength (Figure 4). While the line was observed to be more continuous in non-diabetic dentin groups, spacing of the bond line was observed in diabetic dentins. In addition, spacing was less frequently observed in Variolink N resin cement groups compared to Panavia SA Universal resin cement groups.

## 4. Discussion

The present investigation aimed to assess the influence of different resin cements and CHX on the root dentin bonding of diabetic and non-diabetic teeth. Two different resin cements (Variolink N and Panavia SA) and two different irrigation solutions (CHX and saline) were studied in vitro on diabetic and non-diabetic teeth using a push-out bond strength test. Our findings demonstrated that both the type of resin cement and the condition of the teeth (diabetic or non-diabetic) significantly influenced the bond strength of fiber posts. Consequently, the null hypothesis was rejected.

Diabetes mellitus is one of the factors that can cause endodontic failure [20]. DM is a disease that impairs numerous immune system processes, inhibits the immune system, delays wound healing, lowers the ability to repair damaged tissue, and results in persistent inflammation and progressive tissue degradation [21,22]. Additionally, it lowers the likelihood that the teeth of diabetic patients with preoperative peri-radicular lesions will benefit from root canal therapy [23].

Diabetic patients whose teeth are receiving endodontic treatment should anticipate an increase in the quantity and size of peri-radicular lesions. Thus, the success of the root canal treatment and the selection of resin cements are very important in patients with diabetes mellitus. The success of resin restorations relate to the achievement of a strong and durable bond between the resin cement, fiber posts, and tooth structure [24].

In this study, PBS (considered a reliable method in radicular dentin) was performed to assess the bond strength of different posts to intra-radicular dentin of single root central teeth with diabetes mellitus and non-diabetic teeth [25]. The push-out bond strengths were evaluated in different thirds and also only in the middle third in some studies [26]. In this study, only the middle third of the root was assessed to determine the push-out strength.

In recent years, resin cements have frequently been used in post-endodontic restorations. In the literature, a new classification was reported about resin cements named ‘universal resin cements’; they must have some features noted as containing 10-MDP monomer, dual-cure polymerization, and no need for any adhesive procedures [27]. In the present study, Variolink N, which has multi-step application, and also etch-and-rinse resin cement and Panavia SA Universal, categorized as a ‘Universal Resin Cement’, were investigated for diabetic and non-diabetic teeth. The effect of chlorhexidine on the PBS of these resin cements in root dentin was also investigated.

The diameter and density of tubules are larger in diabetic patients than in non-diabetic patients, and tubule diameters are significant factors in the depth of bacterial invasion [4]. More debris may be present in tubules that are too far away for irrigation solutions and chemo-mechanical preparations to reach during root canal treatment, which could account for the higher failure rate in endodontic treatment success and PBS of resin materials (such as resin-based sealers and resin cements) at cementations. Another factor in endodontic treatment is the bond strength of different root canal sealers and also the bond strength of resin cements if the fiber posts are to be used in post-endodontic restoration of the teeth. In the present study, most of the PBS values of Variolink N groups were significantly higher than the bond strength of Panavia SA cement in both diabetic and non-diabetic teeth, independent of the use of chlorhexidine. Thus, the null hypothesis that the resin cements would not affect the push-out strength of fiber posts in diabetic and non-diabetic teeth was rejected. Variolink N resin cement is a dual-cure resin cement with a total-etch system, and Panavia SA cement is a popular self-adhesive universal cement that does not require any additional steps for bonding. Panavia SA contains 10 MDP, Bis-GMA, and TEGDMA. Information about Panavia SA Universal cement is very limited in the literature [28,29]. According to the results, Variolink N multi-step total-etch resin cement demonstrated the highest push-out bond strength in both groups, compared to Panavia SA, and HEMA could be the reason for the low bond strength. Although HEMA has an important role in increasing the penetration of resins into dentin after demineralization, it has also been reported that HEMA has negative effects on the polymerization of adhesives, the mechanical properties, and the bond strengths. In addition, HEMA causes the hybrid layer to hydrolytically deteriorate over time due to its high water retention, and our results are consistent with this information [30,31].

Diabetes mellitus alters the salivary pH, increases the accumulation of plaque on the restoration surface, and ultimately affects the resin–tooth bond, which causes recurrent caries. Thus, coronal restoration is as important as endodontic treatment. When coronal restorations have microleakage, root canal treatment can be more important in diabetic patients. Matrix metalloproteinases (MMPs) are released at the dentin–adhesive interface associated with the breakdown of exposed collagen [32]. Several studies have focused on using matrix metalloproteinase (MMPs) inhibitors to counteract enzymatic biodegradation in the weak resin-infiltrated hybrid layer to enhance the quality of the resin–dentin interface [18,32,33]. Ng et al. [18] evaluated the effect of MMP inhibitors on the bond strength of resin cements and fiber posts to dentin, reporting that CHX provides a higher push-out bond strength (PBS) compared to EDTA. In this study, CHX had a favorable effect on the push-out strength of self-adhesive universal and total-etch resin cements, consistent with these studies [34]. The results explain that CHX enhances the bond strengths of both resin cements in non-diabetic and diabetic teeth. Chlorhexidine can be adsorbed by dentin, which can infiltrate the resins into the tubules. Therefore, this can explain the high-bond-strength results of the groups with CHX in the present study. The beneficial properties of CHX, including its strong binding ability to phosphate groups, high affinity for enamel and dentin surfaces, and its ability to increase the surface free energy of these tissues, suggest that its application after dentin acid etching may enhance the dentin-wetting ability of primers, thereby improving adhesion [35]. CHX cannot dissolve organic tissue, biofilm, or the smear layer [36,37]. Naenni et al. [38] concluded in a study that none of the solutions, except NaOCl, dissolved necrotic tissue. For these reasons, although CHX is not used alone as an irrigation solution, it is recommended to use it as a final irrigation solution due to its binding to dentin and long-lasting antibacterial effects. A study by Dinesh et al. [39] concluded that the use of CHX as the final irrigation solution increased the bond strength of different root canal filling materials to dentin. In addition, the current study reported decreased peritubular dentin, increased dentin tubule density, and structural alterations in the calcification of the pulp–dentin complex in diabetic teeth [4]. Taking into account the structure of diabetic dentin, these findings corroborate the observation of a reduced push-out bond strength in diabetic teeth. Applying CHX can improve the bond strength of root dentin when it is used before the cementation of fiber posts with total-etch and self-adhesive resin cements because of these reasons.

Variolink N is a multistep total-etch dual-cure resin cement. The highest bond strength of Variolink N was demonstrated by the non-diabetic/CHX group. It has been reported that total-etch resin cements have better bonding strength to dentin, and also self-adhesive resin cements have lower bond strength than conventional resin cements [40]. In this study, Variolink N resin cement exhibited a higher push-out bond strength than the self-adhesive resin cement in all groups, and the use of CHX could improve this bond strength. Adhesive systems in total-etch resin cement can provide a better interaction with dentin than self-adhesive cement, due to the infiltration of the bonding agent into the substrate and the formation of a hybrid layer [41]. Syntac Primer and Syntac Adhesive contain water, which can provide moisture for dentin for better bonding.

In diabetic dentin, for the Panavia SA group, where the post space was rinsed with saline only, SEM images revealed areas of discontinuity at the bonding interface. In contrast, in the non-diabetic dentin group treated with 2% chlorhexidine as the final rinse, SEM images revealed the continuity of the hybrid layer, which is considered a hallmark of optimal bonding interface integrity in the Variolink N group. This result is consistent with the push-out bond strength in groups. In the Variolink N/CHX group, a continuous bonding surface can be observed in Figure 4a; however, cracks were observed in the Variolink N/saline group (Figure 4e).

One limitation of this study is that thermal aging was not performed; aging can be effective in the bond strength of teeth, especially for the application of CHX, because of MMP inhibition [42]. Moreover, in vivo conditions were not imitated, and these could be effective for tooth restoration, allowing for considerations such as mechanical loading while chewing.

Another limitation of this study is that the microhardness of dentin tissue is influenced by factors such as age, gender, and systemic diseases. Systemic complications increase with the duration and extent of high glucose levels [43]. Therefore, the wide age range of diabetic patients from whom the teeth included in the study were obtained, the uncertainty of the accuracy of the diabetes duration reported by the patients, the irregularity of 6-month check-ups, and the unclear data regarding fasting blood glucose and glycated hemoglobin (hemoglobin A1C) are significant limitations. Additionally, since the samples were collected at different time points based on availability, the storage duration of the samples might affect the final results.

## 5. Conclusions

The irrigation of post spaces with CHX enhances the bond strength of adhesive cements in both diabetic and non-diabetic dentin. Notably, in diabetic dentin, CHX irrigation and the use of Variolink N as an adhesive significantly increases bond strength, whereas irrigation with saline yields the lowest values, making it unsuitable for use in diabetic dentin. Additionally, Variolink N demonstrates superior bond strength compared to Panavia SA Universal. Further laboratory studies are necessary to improve the bond strength of resins to diabetic dentin.

## Figures and Tables

**Table 1:** Materials, composition, manufacturers, and their applications.

Material	Manufacturer	Composition	Application
Cytec Blanco	Hanner-Kratt, Germany	60% glass fiber, 40% epoxy resin matrix.	
Total Etch	Ivoclar Vivadent, Schaan, Liechtenstein	37% phosphoric acid.	The post spaces were etched using Total Etch for 15 s, and dried with an air–water spray after rinsing.
Syntac Primers	Ivoclar Vivadent, Schaan, Liechtenstein	Triethylene glycol dimethacrylate, polyethylene glycol dimethacrylate, maleic acid (3–<10%), acetone 25–50%, water.	Syntac Primers were applied for 15 s, and thinned with air.
Syntac Adhesives	Ivoclar Vivadent, Schaan, Liechtenstein	Polyethylene glycol dimetharylate 3–<10% glutaraldehyde, water.	Syntac Adhesives applied for 10 s, and also thinned with air.
Heliobond	Ivoclar Vivadent, Schaan, Liechtenstein	Bis-GMA, dimethacrylate, initiators, stabilizers.	Post spaces were then pretreated with Heliobond.
Variolink N	Ivoclar Vivadent, Schaan, Liechtenstein	Bis-GMA, urethane dimethacrylate, triethylene glycol dimethacrylate, barium glass, ytterbium trifluoride, Ba-Al fluorosilicate glass, spheroid mixed oxide, initiators, stabilizers, and pigments.	Fiber posts were cemented with Variolink N and polymerized for 20 s using an LED.
Panavia SA Universal	Kuraray Noritake Dental Inc., Okayama, Japan	Bis-GMA, TEGDMA, HEMA, MDP, hydrophobic aliphatic dime-thacrylate, hydrophobic aromatic dimethacrylate, silane coupling agent, silanated Ba–glass filler, silanated colloidal SiO_2_, Al_2_O_3_ filler, surface-treated NaF (<1%), CQ, peroxide, catalysts, accelerators, pigments.	Fiber posts were cemented with Panavia SA Universal and polymerized for 20 s using an LED.

**Table 2:** Evaluation of bond strength according to tooth tissue, resin cement, and post-space irrigation solution groups (* *p* < 0.05).

		Non-Diabetic	Diabetic	
Resin Cement	Post-Space Irrigation	Mean ± SD	Mean ± SD	*p*
Variolink N	Saline	9.29 ± 0.66	5.66 ± 0.66	0.001 *
	Saline + Chlorhexidine	14.53 ± 0.58	9.02 ± 0.51	0.001 *
	*p*	0.001 *	0.001 *	
Panavia SA Universal	Saline	6.18 ± 0.32	3.33 ± 0.28	0.001 *
	Saline + Chlorhexidine	8.21 ± 0.63	5.42 ± 0.79	0.001 *
	*p*	0.001 *	0.001 *	
Saline	Variolink-Panavia *p*	0.001 *	0.001 *	
Saline + Chlorhexidine	Variolink-Panavia *p*	0.001 *	0.001 *	

## Data Availability

The original contributions presented in the study are included in the article, further inquiries can be directed to the corresponding authors.
